# From Prion Diseases to Prion-Like Propagation Mechanisms of Neurodegenerative Diseases

**DOI:** 10.1155/2013/975832

**Published:** 2013-10-10

**Authors:** Isabelle Acquatella-Tran Van Ba, Thibaut Imberdis, Véronique Perrier

**Affiliations:** ^1^Université Montpellier 2, 34095 Montpellier, France; ^2^Inserm, U710, 34095 Montpellier, France; ^3^EPHE, 75007 Paris, France

## Abstract

Prion diseases are fatal neurodegenerative sporadic, inherited, or acquired disorders. In humans, Creutzfeldt-Jakob disease is the most studied prion disease. In animals, the most frequent prion diseases are scrapie in sheep and goat, bovine spongiform encephalopathy in cattle, and the emerging chronic wasting disease in wild and captive deer in North America. The hallmark of prion diseases is the deposition in the brain of PrP^Sc^, an abnormal **β**-sheet-rich form of the cellular prion protein (PrP^C^) (Prusiner 1982). According to the prion hypothesis, PrP^Sc^ can trigger the autocatalytic conversion of PrP^C^ into PrP^Sc^, presumably in the presence of cofactors (lipids and small RNAs) that have been recently identified. In this review, we will come back to the original works that led to the discovery of prions and to the protein-only hypothesis proposed by Dr. Prusiner. We will then describe the recent reports on mammalian synthetic prions and recombinant prions that strongly support the protein-only hypothesis. The new concept of “deformed templating” regarding a new mechanism of PrP^Sc^ formation and replication will be exposed. The review will end with a chapter on the prion-like propagation of other neurodegenerative disorders, such as Alzheimer's and Parkinson's disease and tauopathies.

## 1. The Story of the Prion Protein That Was Mistaken for a Virus

Prion diseases and prion infectious agents [1] are among the most fascinating biological topics of the twentieth century and have been under the spotlight for the last 30 years, particularly due to the striking epidemic of bovine spongiform encephalopathy (BSE), which started in Great Britain in the mid-eighties and then spread to other European countries [2]. The transmission of the bovine prion agent to humans, possibly through consumption of prion-contaminated beef products, led to the emergence of a new human prion disease, named “variant” Creutzfeldt-Jakob disease (vCJD), in young people [3]. Recently, several cases of secondary human-to-human transmission of vCJD through transfusion of prion-contaminated blood [4–6] have raised doubts within the scientific community about the safety of blood products and highlighted the crucial need of diagnostic tests for prion detection in blood. Currently, the development of reliable blood tests and of therapies is the main mission of scientists working in the prion field.

Historically, the infectious agent that causes prion diseases was supposed to be an atypical virus belonging to the category of “slow viruses” [7, 8]. Then, in 1967, Pattison and colleagues reported [9] that the scrapie agent was resistant to heat and formaldehyde, two treatments that inactivate most viruses, thus introducing a doubt about the true nature of this infectious agent. In addition, in 1967, Alper and colleagues showed that the scrapie agent was also resistant to ionizing radiations and UV light irradiation that normally inactivates nucleic acids, suggesting that it was probably devoid of nucleic acids [10]. Based on these intriguing experimental data, Griffith suggested that the scrapie agent could be a protein that self-replicates through autocatalytic conformational changes [11]. This audacious hypothesis retained the attention of Stanley Prusiner who purified the scrapie agent from the brain of scrapie-infected hamsters and reported that inactivation by physicochemical agents that destroy proteins abolished the infectivity of such purified preparations [12]. In 1982, he proposed the new term of “PRION,” for “proteinaceous infectious only particle,” to define this atypical agent. The revolutionary idea that a protein may act as a virus was unbelievable at that time and Stanley Prusiner had to struggle hard to convince the scientific community. His outstanding work on prions earned him the Nobel Prize of Medicine in 1997, although at that time the ground-breaking concept of proteinaceous infectious particles was not yet definitively proved.

## 2. Prion Diseases: The Revolutionary Concept of Pathogenic Misfolded Proteins

The prion protein (PrP) is the main component of prion agents and, remarkably, can fold into different (normal or pathogenic) conformations that are thermodynamically stable [13]. PrP^C^, the normal cellular isoform, is mostly folded into *α*-helices [14] and is detergent-soluble and completely digested by proteinase K. Conversely, PrP^Sc^ (for scrapie form), the abnormally folded isoform, is mostly folded into *β*-sheets [15], which confer insoluble property in detergents, and is partially resistant to proteinase K. Indeed, PrP^Sc^ digestion by proteinase K produces an N-terminally truncated fragment that begins around residue 90 and is commonly called PrP27-30. PrP^Sc^ isoforms are the main constituent of amyloid plaques and of brain deposits in patients affected by CJD. For this reason, PrP^Sc^ is considered as the main disease marker and is the reference for the histopathological analyses carried out to diagnose prion diseases. 

**Figure 1:**
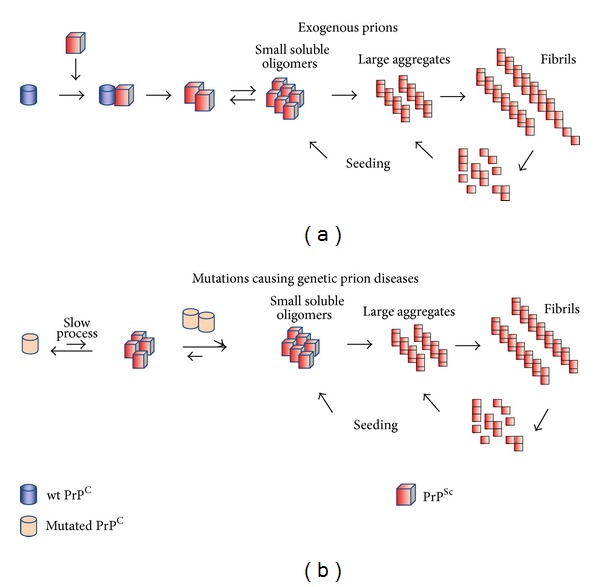
Proposed mechanisms of PrP^C^ conversion into PrP^Sc^, with exogenous (a) or genetic (b) prions. Wild-type PrP^C^ is represented by a cylinder colored in blue, mutated PrP^C^ is outlined in blue, and PrP^Sc^ is represented by red square.

How can prions multiply? In the original “protein-only” hypothesis proposed by Griffith and Prusiner [1, 11], PrP^Sc^ can trigger the autocatalytic conversion of normal PrP^C^ into PrP^Sc^ and imprints its misfolded form to PrP^C^, which in turn becomes pathological (Figure 1). This conversion process involves several PrP^Sc^ intermediates that are generated through a complex oligomerization mechanism and then self-assembled into protofibrils, which in turn grow into amyloid fibrils [16, 17]. Then, large fibrils can break naturally, producing small fragments, called seeds, that will propagate *de novo* the prion agent (seeding process) [18–20]. As both PrP^C^ and PrP^Sc^ are exposed at the cell surface and attached to the plasma membrane through a GPI anchor, they can propagate in tissues via cell-cell contacts [21, 22]. Many recent lines of evidence indicate that the most neurotoxic species within this replication cycle are the small soluble oligomers rather than the large amyloid fibrils, which would serve as “reservoirs” to trap small neurotoxic species [17, 23, 24].

## 3. Development of Animal Models to Study Prion Infectivity

The advent of molecular biology allowed the generation of cell (the neuroblastoma ScN2a cell line) and transgenic animal models [25–27] to investigate the molecular basis of prion replication, pathogenicity, and propagation. The crucial role of PrP was demonstrated using mice in which the gene coding for PrP (*Prnp*) was genetically ablated [28, 29]. These mice are resistant to prion inoculation and cannot propagate and replicate the infectious agent. Later on, *Prnp *knock out (using the *cre/lox* system) in the neurons of adult mice with early prion infection allowed demonstrating that the synaptic impairment, spongiosis, and behavioural deficits observed in these animals could be reversed [30]. Conversely, transgenic mice that harbour high copy numbers of a wild-type *Prnp* transgene develop a neurological syndrome that is similar in some aspects to prion disease, but they do not produce transmissible PrP^Sc^ unless they are inoculated with prions [31]. For decades, no animal model of sporadic prion disease was available in which prions formed spontaneously from wild-type PrP and could be transmitted to other animals. Interestingly, spontaneous development of transmissible spongiform encephalopathy was observed in transgenic mice that overexpress a mouse-elk PrP chimeric molecule harbouring the two point mutations S170N and N174T that induce a rigidity of the *β*2-*α*2 loop region [32]. The disease could be transmitted by intracerebral inoculation of brain homogenates from ill mice to tga20 mice that overexpress wild-type PrP and from them to wild-type mice. These findings illustrate the importance of PrP *β*2-*α*2 loop region. This region is rich in glutamine and asparagine residues, which are frequently encountered in amyloidogenic proteins, and may act as “hot spots” for protein aggregation. Recently, Watts et al. generated a transgenic mouse model, named Tg(BVPrP), that overexpresses wild-type bank vole (BV) PrP. These mice develop spontaneous CNS dysfunction between days 108 and 340 after birth that recapitulates the hallmarks of prion diseases [33]. Moreover, the disease could be transmitted to tga20 and wild-type mice by intracerebral injection of brain homogenates from ill Tg(BVPrP) animals. This is the first animal model showing that wild-type PrP can spontaneously form infectious prions *in vivo* and thus will be very useful for understanding the aetiology of sporadic prion diseases, such as sporadic CJD.

Several transgenic mice that overexpress the most frequent mutant PrP proteins, such as P101L PrP [34, 35] or PrP with a 9 octarepeat insertion in the N-terminus [36], were also generated. Although these mice succumb to spontaneous prion disease with various incubation times, they do not show all the biochemical and pathological features of prion diseases and often fail to transmit prion infectivity to wild-type animals. Recently, several transgenic mouse models of genetic prion diseases retained our interest:

- the Tg(PG14) transgenic mice that express a mutant PrP with 14 octapeptide repeats [37] and present a progressive neurological disorder with ataxia, PrP deposition, and massive loss of cerebellar granule cells. They also display the main biochemical properties of PrP^Sc^, such as partial resistance to proteinase K, detergent insolubility and resistance to GPI-anchor cleavage by phospholipase;
- the Tg(MHu2ME199K) mouse model of genetic CJD, which is caused by the E200K substitution in human PrP. These animals express a chimeric mouse-human PrP harbouring the corresponding mouse E199K mutation (PrPMHu2ME199K) [38] and develop progressive neurodegenerative disease from 6 months of age. Histopathological analysis of their brain revealed the presence of astrocytic gliosis, spongiosis, and PK-resistant PrP deposits by western blotting. Importantly, brain extracts from Tg(MHu2ME199K) sick mice transmitted the prion disease to wild-type animals [38];
- the knock-in Ki-3F4-FFI mice that express a mutant PrP (D177N-M129-3F4 tagged) associated with fatal familial insomnia (FFI), a genetic human prion disease [39]. These mice present several neurological features (atrophied cerebellum, enlarged ventricles, and thalamus abnormalities) that are similar to those seen in humans with FFI. Surprisingly, these animals display a protease-sensitive PrP (sPrP) isoform, like patients with FFI, and the disease is transmissible to control Ki-3F4-WT mice (wild-type *Prnp*) and to transgenic mice that overexpress wild-type PrP (tga20) [40]. The presence of PK-sensitive PrP^Sc^ in FFI mice supports recent findings showing that new PK-sensitive synthetic prions can be infectious [41], as well as the identification of a novel human prion disease called VPSPr (variably protease-sensitive prionopathy) [42], which is characterized by the presence of PrP^Sc^ with highly variable PK resistance. As for all neurodegenerative disorders, it is important to generate the most appropriate animal models of the genetic forms of the disease in order to develop pertinent therapeutic strategies.

## 4. Synthetic Mammalian Prions and Recombinant Prions: The Proof-of-Concept of the Protein-Only Hypothesis

**Figure 2:**
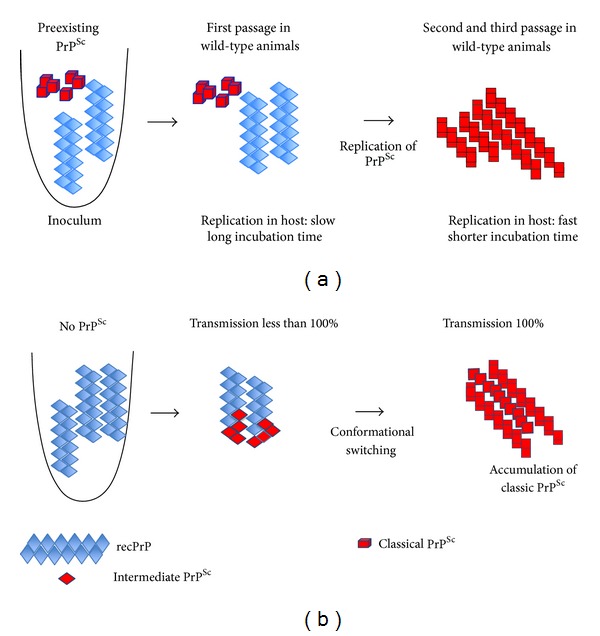
Schematic representation of two possible mechanisms of *de novo* propagation of prions from recPrP fibrils. (a) In this model, the preparation of recPrP fibrils contains very small amounts of classical PrP^Sc^. A long incubation time is required to amplify and propagate *in vivo *this minute amount of PrP^Sc^. (b) The second model (“deformed templating" mechanism) hypothesizes that there is no classical PrP^Sc^ in the fibril preparation and that recPrP fibrils can be converted into PrPres (PrP^Sc^-like structures) with low efficiency. After several passages, these PrP^Sc^-like structures progressively adopt the structural features of classical PrP^Sc^. Schema adapted from Makarava et al., 2011 [48].

Despite the compelling evidence in favour of the prion hypothesis, some sceptics argued that the definite proof could be obtained only by producing *in vitro* the infectious material used for intracerebral inoculation starting from pure normal PrP, a technical feat that seemed impossible for many decades. A strong advance was the finding that recombinant hamster PrP(90-231) (recPrP, which corresponds to human PrP27-30) purified from *E. coli *under reducing conditions at pH > 7 has a high *β*-sheet content and low solubility, like PrP^Sc^. Conversely, recPrP refolding by oxidation to form a disulphide bond produced a soluble protein with a high *α*-helix content, similar to normal PrP^C^ [43]. The ability of recPrP to adopt either an *α*-helix- or *β*-sheet-rich conformation strongly suggests that the PrP sequence is intrinsically plastic. Some PrP domains may have a relatively open conformation which makes it susceptible to conversion into PrP^Sc^ under appropriate physicochemical conditions [44]. However, inoculation of the *β*-sheet-rich recPrP isoform in mice did not transmit the disease. At the beginning of 2000, Baskakov et al. [19, 20] managed to partially solve the complex pathway of PrP assembly into amyloids. To study the kinetic pathway of amyloid formation, they used an unglycosylated recombinant PrP form that corresponds to the PK-resistant core of PrP^Sc^ and found that it can adopt two abnormal *β*-sheet-rich isoforms (*β*-oligomers and amyloid fibrils) via separate kinetic pathways. The tendency to generate either form is driven by the experimental conditions. Acidic pH (similar to the pH found in endocytic vesicles) favours the transition from *α*-monomers to *β*-oligomers, whereas neutral pH promotes amyloid fibril formation [20]. These multiple misfolding pathways and the generation of distinct *β*-sheet-rich isoforms might explain the difficulties to generate infectious prions *in vitro* from pure recombinant PrP. Then, Baskakov and Legname inoculated some amyloid fibrils from purified recombinant PrP(89-231) in the brain of Tg(PrP89-231) transgenic mice that express a truncated PrP variant corresponding to the PK-resistant core. After 580 days of incubation, all injected mice were sick and showed neurological symptoms reminiscent of prion diseases. Analysis of brain tissue sections revealed spongiosis, astrocytic gliosis, and the presence of PK-resistant PrP^Sc^ [45]. At the second passage, brain extracts from these mice were inoculated to both Tg(PrP89-231) and wt FVB mice. Both types of mice showed clinical signs and the biochemical features of prion disease after 150 days (FVB mice) and 250 days (Tg(PrP89-231) animals) of incubation. These findings indicate that a new prion strain can be generated from pure recombinant PrP designated “synthetic mammalian prions” and that it can induce a transmissible neurodegenerative disease in transgenic mice. Subsequent *in vivo* experiments with various synthetic prion strains obtained from recombinant PrP fibrils demonstrated that conformationally stable recombinant amyloids produced more stable prion strains with a longer incubation time, whereas more labile amyloids generated less stable strains with a shorter incubation time [46]. One major criticism to this work is that the recombinant fibrils were first injected in transgenic animals that overexpress PrP and not in wild-type mice. Thus, the inoculation of recombinant fibrils might have resulted in an acceleration of preexisting conditions produced by transgenesis as it is the case for transgenic mice that overexpress normal or mutated PrP [31, 36]. However, injection of recombinant hamster PrP (recPrPHa) fibrils in wild-type Golden Syrian hamsters provided strong evidence that fibrils can induce transmissible disease *de novo* [47], although 100% success rate was only achieved at the second passage and was correlated with the presence of PrP^Sc^ in the brain. The animals showed clear signs of transmissible spongiform encephalopathy (TSE), and the unique clinical course and neuropathological features suggested that a new prion disease was induced by recPrPHa fibrils. This new prion strain was designated as SSLOW due to the very long disease incubation time. These experiments are in strong favour of the protein-only hypothesis; however, it remains to be elucidated how recombinant PrP fibrils trigger the formation of transmissible PrP^Sc^. The predominant hypothesis, which is based on the “template-assisted” mechanism of propagation, is that the preparation of recombinant PrP fibrils might have included some minute amount of PrP^Sc^ (Figure 2(a)) that was responsible for the disease. This could also explain the long incubation time and the lower than 100% transmissibility at the first passage. However, recent work by Makarava et al. [48, 49] suggests a new templating mechanism, called “deformed templating” (Figure 2(b)). Three different inocula with conformationally distinct amyloid states (0.5 M fibrils, 2 M fibrils and S fibrils) were prepared *in vitro* from purified recPrPHa [49]. After inoculation in mice, no signs of prion infection were found in animals injected with 2 M and S fibrils that are reminiscent of PrP^Sc^, whereas the less stable 0.5 M fibrils induced a pathogenic process that eventually led to transmissible prion disease. Using the protein misfolding cyclic amplification (PMCA) technique, they showed that the 0.5 M recPrPHa fibrils used to inoculate wild-type animals did not contain classical PrP^Sc^. However, these fibrils gave rise to an atypical proteinase K-resistant PrP (PrPres) that was detected using a modified PMCA procedure. This atypical transmissible PrPres has a structure that resembles that of amyloid seeds and was observed during the asymptomatic stage of the disease before the emergence of the classical PrP^Sc^ form [49]. This work provides evidence that apparently noninfectiou amyloid fibrils with a structure different from that of PrP^Sc^ can lead to transmissible prion disease and suggests a new mechanism of prion conversion through “deformed templating.” In this model (Figure 2(b)), recombinant PrP fibrils, which have a structure that is significantly different from that of PrP^Sc^, can progressively acquire a new folding pattern and adapt to the template of the classical PK-resistant PrP^Sc^ [48, 49].

An alternative approach to demonstrate the protein-only hypothesis was explored by Wang et al. [50] who created recombinant prions by PMCA using recombinant mouse PrP (purified from *E. coli*) in the presence of synthetic phospholipids and total liver RNA. The recombinant prions obtained in these conditions showed all the features of the pathogenic PrP isoform, especially the protease resistance and transmissibility in wild-type CD-1 mice that succumbed to prion disease in about 150 days. This experiment provides strong evidence in support of the protein-only hypothesis because prions with high infectivity titre could be generated *in vitro* from well-defined components. It also illustrates the key role of lipids and RNA as cofactors to facilitate PrP conversion. Recently, Deleault et al. identified phosphatidylethanolamine as the single cofactor required to facilitate the conversion of recombinant PrP into infectious recombinant PrP^Sc^ during PMCA [51, 52].

## 5. Extending the Prion Concept to Other Neurodegenerative Diseases: The Prionopathy World

During the last decades, many publications have shown that neurodegenerative disorders as diverse as Alzheimer's, Parkinson's, Huntington's, and Creutzfeldt-Jakob disease share a common pathogenic mechanism involving the aggregation and deposition of misfolded proteins. Although the type of aggregated proteins is disease specific (Table 1), they all share a “prion-like” mechanism of cell-cell propagation, with similar pathways of protein aggregation that involve oligomeric species leading to fibril formation and amyloid deposition. For instance, several studies have investigated the putative prion-like mechanism involved in the transmission of misfolded amyloid beta (A*β*) (Table 1) by inoculating brain extracts from patients with Alzheimer's disease (AD brain tissues) in several animal models. In marmoset, a non-human primate, amyloid plaques were induced 6-7 years after inoculation of AD brain tissue [53]. These plaques were composed of aggregated A*β* peptides similar to those found in the host, and A*β* deposition was not restricted to the injection site, suggesting diffusion of the newly formed aggregates. The experiment could be successfully reproduced in Tg2576 transgenic mice that express the *β*-amyloid precursor protein (APP) with the Swedish mutation corresponding to the familial form of AD. Specifically, intracerebral injection of AD brain tissue in these animals led to a peculiar brain distribution of A*β* deposits [54, 55]. Five months after injection, the A*β* aggregates were localized only in the ipsilateral side, whereas after 12 months senile plaques and vascular deposits were detected in both hemispheres, suggesting spreading of the aggregates. The use of other transgenic mouse models (APP23 and APP/PS1 animals) [56] showed that the brain A*β* deposit profiles vary with the host and the brain extracts used to induce amyloidosis, similar to what was observed with different prion strains [56–58]. Altogether these results clearly indicate that inoculation of brain extracts containing preformed A*β* seeds accelerates the formation of new A*β* deposits *in vivo*, in transgenic mice and non-human primates. They also support the hypothesis of a transmissible origin of AD. Remarkably, Stohr and coworkers induced cerebral *β*-amyloidosis by inoculating purified A*β* aggregates derived from brain or aggregates composed of synthetic A*β* peptides in Tg(APP23:*Gfap*-luc) mice [65]. Monitoring of A*β* deposition in live Tg(APP23:*Gfap*-luc) mice by using bioluminescence imaging showed that A*β* aggregates self-propagate as prions.

Similar findings were reported concerning the induction of Tau aggregates [59] in the brain of transgenic mice that express wild-type human Tau after intracerebral injection of brain extracts from old Tg(HuTauP301S) mice containing insoluble Tau aggregates. Neurofibrillary tangles, neuropil threads, and coiled bodies could be visualized not only in neurons but also in oligodendrocytes of the injected animals. In addition, mouse Tau can coaggregate with human Tau P301S, indicating cross-species seeding [60].

The hallmark of Parkinson's disease (PD) is the presence in the brain of Lewy bodies and Lewy neurites that contain high amounts of aggregates of misfolded *α*-Synuclein (*α*-Syn). In the dual-hit hypothesis proposed by Hawkes and coworkers, PD originates in the nose and foregut after inhalation/ingestion of an unknown neurotropic pathogen and then aggregates spread throughout the nervous system with a stereotypic pattern following unmyelinated axons [66]. This theory is based on extensive postmortem analyses of patients with PD that identified the olfactory bulb and enteric plexus of the stomach as early sites of Lewy pathology, and also on evidence of olfactory and autonomic dysfunction as early nonmotor PD symptoms [66]. Based on this hypothesis, Luk et al. [63] have stereotaxically injected preformed recombinant *α*-Syn fibrils in the cortex and striatum of Tg(*α*-SynA53T) mice that express human *α*-Syn harbouring the A53T mutation related to familiar PD and showed that *α*-Syn aggregates can spread in the tissues with a prion-like mechanism of propagation. Similarly, a single intrastriatal inoculation of synthetic *α*-Syn fibrils in wild-type nontransgenic mice led to the cell-to-cell transmission of pathologic *α*-Syn and Parkinson's-like Lewy pathology in anatomically interconnected regions. Accumulation of toxic aggregates in these mice triggered a progressive loss of dopamine neurons in the *substantia nigra* and a reduced dopamine levels culminating in motor deficits [64]. Remarkably, experiments of transplantation of fetal cells in PD subjects showed the presence of Lewy body-like inclusions 14 years after grafting that stained positively for *α*-Syn and ubiquitin and had reduced immunostaining for dopamine transporter [61]. This result was confirmed in other PD subjects transplanted with fetal mesencephalic dopaminergic neurons (11–16 years) who developed *α*-Syn-positive Lewy bodies in grafted neurons [62]. These results suggest a host-to-graft disease propagation mechanism with implications for cell-based therapies [61, 62].

## 6. Conclusion

These last years have been marked by the end of the controversy about the protein-only hypothesis concerning prion diseases. In addition, a growing number of studies have shown that other amyloidogenic proteins implicated in various neurodegenerative disorders can propagate *in vivo* with a prion-like mechanism. We witness the opening of a new field of research in neurodegenerative disorders [67, 68], and the lessons learned from prion diseases will help scientists develop new strategies for diagnostic and therapeutic approaches for other neurodegenerative disorders.

## Figures and Tables

**Table 1:** Prion model of induction described for neurodegenerative diseases.

Disease	Normally folded protein “Precursor”	Abnormally folded protein “Prion form”	Protein aggregates detected	Seeding inoculum	Prion-like propagation in mammals	References
CJD/scrapie	PrP^C^	PrP^Sc^	PrP^Sc^ deposits plaques	Various mammalian prions and recPrP fibrils	WT and Tg mice Non-human primates	[32, 33, 37–40] [45–50]

Alzheimer (AD)	Amyloid precursor protein (APP)	Amyloid beta peptides A*β*	A*β* plaques	Human AD and Tg mice brain extracts blood	Marmosets TgAPP2576 TgAPP23, TgAPP/PS1	[53–58]

Tauopathies	Tau	Tau aggregates	Neurofibrillary tangles (NFTs)	Tg(HuTauP301S) brain extracts	Tg(wt Tau)	[59, 60]

Parkinson (PD)	*α*-Synuclein	*α*-Synuclein aggregates	Lewy bodies	Human preformed *α*-Syn fibrils	(i) Fetal tissue grafts in human PD patients	[61, 62]
					(ii) Tg (*α*-SynA53T) and WT mice	[63, 64]
